# Activation of the Renin–Angiotensin–Aldosterone System Is Attenuated in Hypertensive Compared with Normotensive Pregnancy

**DOI:** 10.3390/ijms241612728

**Published:** 2023-08-12

**Authors:** Robin Shoemaker, Marko Poglitsch, Hong Huang, Katherine Vignes, Aarthi Srinivasan, Cynthia Cockerham, Aric Schadler, John A. Bauer, John M. O’Brien

**Affiliations:** 1 Department of Dietetics and Human Nutrition, University of Kentucky, Lexington, KY 40506, USA; 2 Attoquant Diagnostics GmbH, 1110 Vienna, Austria; 3 Department of Pediatrics, University of Kentucky, Lexington, KY 40536, USA; 4 Division of Maternal Fetal Medicine, Department of Obstetrics and Gynecology, University of Kentucky, Lexington, KY 40506, USA

**Keywords:** angiotensin, maternal, pregnancy hypertension, biomarkers, mass spectrometry, aldosterone

## Abstract

Hypertension during pregnancy increases the risk of adverse maternal and fetal outcomes, but the mechanisms of pregnancy hypertension are not precisely understood. Elevated plasma renin activity and aldosterone concentrations play an important role in the normal physiologic adaptation to pregnancy. These effectors are reduced in patients with pregnancy hypertension, creating an opportunity to define the features of the renin–angiotensin–aldosterone system (RAAS) that are characteristic of this disorder. In the current study, we used a novel LC-MS/MS-based methodology to develop comprehensive profiles of RAAS peptides and effectors over gestation in a cohort of 74 pregnant women followed prospectively for the development of gestational hypertension and pre-eclampsia (HYP, 27 patients) versus those remaining normotensive (NT, 47 patients). In NT pregnancy, the plasma renin activity surrogate, (PRA-S, calculated from the sum of Angiotensin I + Angiotensin II) and aldosterone concentrations significantly increased from the first to the third trimester, accompanied by a modest increase in the concentrations of angiotensin peptide metabolites. In contrast, in HYP pregnancies, PRA-S and angiotensin peptides were largely unchanged over gestation, and third-trimester aldosterone concentrations were significantly lower compared with those in NT pregnancies. The results indicated that the predominant features of pregnancies that develop HYP are stalled or waning activation of the RAAS in the second half of pregnancy (accompanied by unchanging levels of angiotensin peptides) and the attenuated secretion of aldosterone.

## 1. Introduction

New-onset hypertension during pregnancy (gestational hypertension or pre-eclampsia) affects 5–10% of pregnancies, and the prevalence in the United States is rising [1]. The development of hypertension during pregnancy is associated with long-term health risks for both the mother and the offspring [2], and increases the maternal risk of cardiovascular diseases later in life [3,4]. Knowledge of the mechanisms underlying perinatal hypertension could lead to improved strategies for the treatment and/or management of hypertension during pregnancy [5,6]. 

The renin–angiotensin–aldosterone system (RAAS) is a master regulator of blood pressure and fluid homeostasis in pregnant and non-pregnant states [7,8]. The activity of the RAAS is initiated by the enzymatic cleavage of a 10-amino acid peptide, Angiotensin I (Ang I) from the N-terminus of angiotensinogen (AGT) by the enzyme renin in a highly regulated reaction. The rate of Ang I formation in plasma, also called plasma renin activity (PRA), is determined by the concentrations of active renin and AGT, where renin is rate-limiting. Ang I serves as a substrate for angiotensin-converting enzyme (ACE), resulting in the formation of angiotensin II (Ang II), the primary effector molecule of the RAAS. The concentration of active Ang II is controlled via negative feedback of renin [9], and by metabolism at the N- or C-terminal by amino and carboxy peptidases in the plasma, where the latter is considered to be a counter-regulatory arm of the RAAS. The cleavage of Ang II by Angiotensin-converting enzyme 2 (ACE2) generates angiotensin-1-7 (Ang 1-7) [10], a peptide with purported vasodilatory and other counter-effects to Ang II [11]. 

The classical effects of Ang II are mediated through the Ang II Type 1 (AT_1_) receptor, and include vasoconstriction, activation of the sympathetic nervous system, and stimulation of the release of aldosterone from the adrenal cortex [12,13]. Outside pregnancy, the effects of excess Ang II and aldosterone are associated with inflammation, fibrosis, and endothelial dysfunction [14,15], and abnormal function of the counter-regulatory arm of the RAAS has been described in many cardiovascular [16,17], renal [18], and infectious disease states(e.g., COVID-19) [19]. 

In a healthy pregnancy, there is pronounced activation of the RAAS, where the concentrations of PRA and aldosterone are increased compared with non-pregnant states [20,21,22]. The upregulation of renin activity and aldosterone secretion play key roles in physiologic adaptations to a healthy pregnancy, such as promoting sufficient reabsorption of water and sodium to support the demand for a 40–50% increase in blood volume [8,23,24]. In contrast, numerous studies have demonstrated that pregnancies with pre-eclampsia or gestational hypertension have lower plasma renin activity and lower aldosterone concentrations compared with normotensive pregnancies. [21,25,26,27]. Limited studies suggest the alternative RAAS may also play a role in pregnancy, and that alterations in these components, e.g., Ang-1-7 and/or ACE2, are associated with pre-eclampsia and infants who are small for their gestational age [28,29,30]. The course of the activity of RAAS over the course of gestation is not precisely understood in either normal or hypertensive pregnancies [23,31]. Understanding key deviations in the normal activity of the RAAS in pregnancy could lead to improved detection or management of gestational hypertension and/or pre-eclampsia. 

RAS Fingerprint^TM^ is novel, liquid chromatography-tandem mass spectrometry (LC-MS/MS)-based methodology for simultaneous quantification of multiple components of the RAAS in a single sample. Outside pregnancy, recent studies have utilized RAS Fingerprint^TM^ (or RAS profiling) to generate a biochemical “snapshot” of the RAAS activity that is reflective of the diseased state in patients with hypertension [32], heart failure [33,34], and other diseases [35,36]. In the current study, we used RAS Fingerprint to quantify the key effectors (angiotensin peptides and aldosterone) of the RAAS in a cohort of women followed prospectively for the development of gestational hypertension and pre-eclampsia. Our objective was to define the prominent features of RAAS activity associated with the development of pregnancy hypertension. 

## 2. Results

### 2.1. Subject Characteristics

This was a secondary analysis of pregnant individuals enrolled in an ongoing study at the University of Kentucky. Paired clinical data and biological samples from visits in the first and third trimester were obtained from 74 women between April 2019 and May 2020 by the maternal and fetal medicine clinicians involved in the study as part of routine patient care. In the current study, patients were grouped for analysis based on whether they reached the primary endpoint of developing either pre-eclampsia or gestational hypertension (HYP, n = 27), or remained normotensive (NT, n = 47). The cohort’s demographics, clinical characteristics, and outcomes are described in Table 1. The cohort was primarily Caucasian, with Black, Hispanic, and mixed-race individuals distributed fairly equally between the groups. Overweight/obesity, diabetes, and a history of pre-eclampsia were prevalent among both the NT and HYP group, reflecting the fact that the patient population was recruited from a high-risk clinic. The HYP group had a slightly greater risk profile compared with the NT group, with more patients being primiparous, having had a previous pre-eclamptic pregnancy, having Type 1 diabetes, and taking aspirin for the prevention of pre-eclampsia. The NT group contained slightly more patients with Type 2 diabetes, and those with renal or autoimmune disease. First-trimester BMI was significantly greater in the HYP group compared with the NT group, as was the first- and third-trimester SBP and DBP. The duration of gestation was significantly shorter in the HYP versus NT groups.

The HYP group included 23 patients who developed gestational hypertension (the mean onset was 35.2 gestational weeks) and 6 patients who developed pre-eclampsia (a mean onset of 34.6 gestational weeks). No patients in either group took antihypertensive medications at the start of pregnancy, but by the third trimester, four patients from the HYP group were prescribed labetalol (three) or nifedipine (one) for managing high blood pressure. Both NT and HYP patients experienced adverse outcomes such as gestational diabetes, PPROM, polyhydramnios, and postpartum hemorrhage. Slightly more HYP than NT patients developed gestational diabetes, but no HYP patients had IUGR or pre-term labor/delivery, compared with two and three, respectively, in the NT group. In the NT group, antidiabetic medications (insulin or metformin) were taken by 10 of 12 patients with Type 1 and/or Type 2 diabetes, and 2 of 3 patients with gestational diabetes. In the HYP group, 9 of the 11 patients with Type 1 or Type 2 diabetes, and 3 of the 4 patients with gestational diabetes took antidiabetic agents. There were four patients with Type 1 diabetes with poorly controlled blood sugar, two who remained normotensive, and two who developed gestational hypertension. 

### 2.2. Quantification of PRA-S and Aldosterone: Attenuated Activation of the Classical RAAS over Gestation in Pregnancies That Developed Hypertension versus Those Remaining Normotensive

We used RAS Fingerprint with equilibrium analysis to quantify six angiotensin peptides, aldosterone concentrations, and biomarkers in the serum of pregnant women in the first and third trimesters of pregnancy. The plasma renin activity surrogate (PRA-S) was calculated from the sum of the equilibrium concentrations of Ang I and Ang II, the surrogate for ACE activity (ACE-S) was calculated from the ratio of the equilibrium concentrations of Ang II to Ang I, the aldosterone to Ang II ratio (AA2-R) was calculated by dividing the concentrations of aldosterone by the equilibrium concentrations of Ang II. Table 2 lists the median and IQR for all measured and calculated components of the RAAS in the cohort. In general, most components of the RAAS were significantly increased over gestation in the NT group, but not the HYP group, and tended to be lower in the HYP group versus the NT group in the third trimester. A subanalysis of the HYP group was performed to examine the concentrations of the components of the RAAS in the 23 patients who developed gestational hypertension separately from those (n = 6) who developed pre-eclampsia, and the median values and IQR for each component for these groups are displayed in Supplementary Table S1. There was no significant difference in the concentrations of the components of the RAAS at either time point in those who developed gestational hypertension compared with pre-eclampsia. In contrast to the NT group, no components of the RAAS were significantly increased over gestation in either those who developed gestational hypertension or pre-eclampsia, with the exception of Ang I in the gestational hypertension group: the median (IQR) in the first trimester was 75.5 (40.9–102.4) pmol/L, and in the third trimester, it was 81.9 (55.6–145.1) pmol/L (*p* < 0.05; Supplementary Table S1). The trend of no change in the components of the RAAS over gestation was most evident in the six women who developed pre-eclampsia.

**Figure 1:**
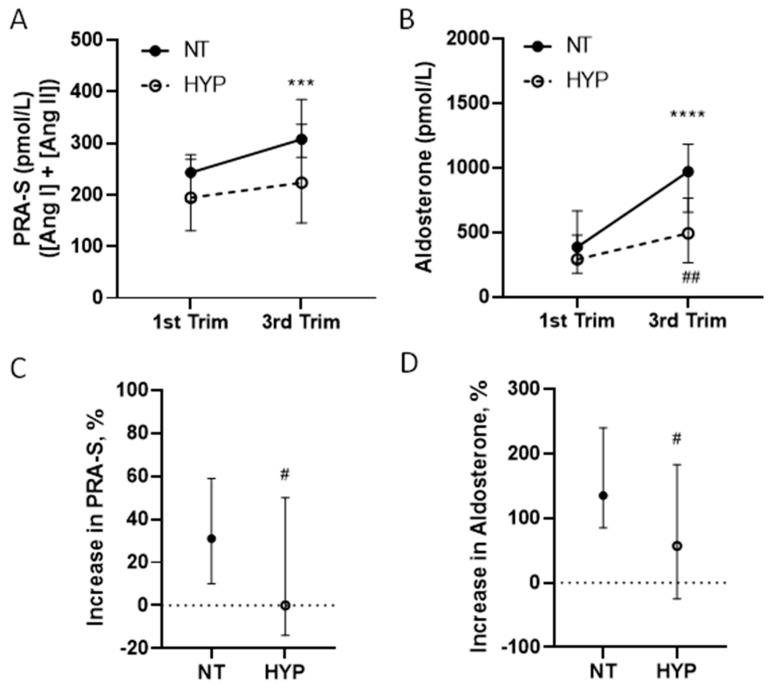
RAS Fingerprint-generated biomarkers of the activation of the classical RAAS in normotensive versus hypertensive pregnancies. (**A**) PRA-S, a surrogate for plasma activity calculated from the sum of the equilibrium concentrations of Ang I and Ang II, and (**B**) concentrations of aldosterone in the first and third trimesters of pregnancies that developed hypertension after 20 weeks of gestation (HYP, n = 27) and those that remained normotensive (NT, n = 47). PRA-S and aldosterone levels were not different among the NT and HYP groups in the first trimester, but increased over gestation only in the group that remained NT. Data are the median and 95th confidence intervals (CI). ***, *p* < 0.001 and ****, *p* < 0.0001 for within-group increases from the first to third trimester analyzed by Wilcoxon’s matched-pairs signed rank tests. ##, *p* < 0.01 compared with NT using the Mann–Whitney test. The percentage of change in (**C**) PRA-S and (**D**) concentrations of aldosterone from the first to third trimester in NT and HYP patients. The percentage of increase over gestation in PRA-S and aldosterone was significantly lower in pregnancies that developed HYP compared with the NT group. Data are the median and 95th CI of the percentage of change for each biomarker over pregnancy in the NT and HYP groups. #, *p* < 0.05 compared with NT using the Mann–Whitney test.

As previous studies of pregnancy have indicated that classical biomarkers, such as activation of the RAAS, PRA (or direct renin concentrations), and plasma aldosterone concentrations (PAC), are reduced in gestational hypertension or pre-eclampsia [25,27,37], we examined the concentrations of PRA-S and aldosterone generated using RAS Fingerprint in our cohort of NT and HYP pregnancies (Figure 1). PRA-S was significantly increased from the first to third trimester in pregnancies that remained NT (*p* < 0.001), with a median increase of 31% (Figure 1A,C). In contrast, PRA-S was not statistically increased in pregnancies that developed HYP (*p* = 0.889), and the median percentage of change in PRA-S over gestation in the HYP group was zero (Figure 1A,C). The median values of PRA-S were not different between the NT and HYP groups at either the first or third trimester, although there was a trend toward a lower PRA-S in the HYP group versus the NT group in the third trimester (*p* = 0.067; Figure 1A, Table 2). Similarly, concentrations of serum aldosterone significantly increased over gestation in the NT group (*p* < 0.0001), with a median increase of 135%. In the HYP group, aldosterone rose by 57%, although the change over gestation was not significant (*p* = 0.141), and the third-trimester concentrations of aldosterone were significantly lower in the HYP group versus the NT group (*p* < 0.01; Figure 1B,D, Table 2). 

### 2.3. Quantification of Angiotensin Peptides: Metabolism of Ang II and Activation of the Counter-Regulatory RAAS in NT and HYP Pregnancies

**Figure 2:**
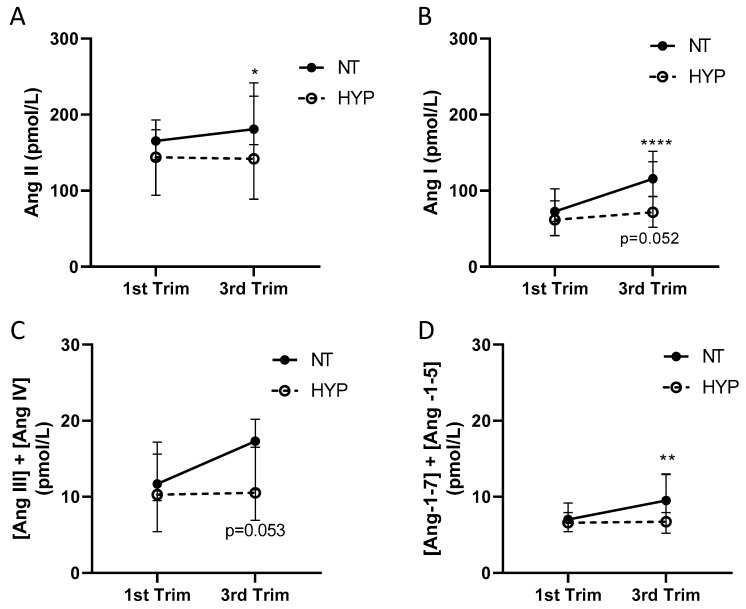
Equilibrium concentrations of Ang II, and upstream and downstream angiotensin metabolites in normotensive and hypertensive pregnancy. (**A**) Ang II and (**B**) Ang I concentrations in the first and third trimesters in the NT (n = 47) and HYP (n = 27) groups. Data are the median and 95th confidence intervals (CI). *, *p* < 0.01, ****, *p* < 0.01 for within-group increases from the first to third trimester analyzed by Wilcoxon’s matched-pairs signed rank tests; *p* = 0.052 for the increase across gestation in HYP patients. (**C**) The concentration of N-terminal metabolites of Ang II, calculated from the sum of the concentrations of Ang III and Ang IV, and (**D**) the concentration of C-terminal metabolites of Ang II, calculated from the sum of the concentrations of Ang-1-5 and Ang-1-7. Data are the median and 95th CI. *p* = 0.053 for the between-group analysis at the third trimester analyzed via the Mann–Whitney test . **, *p* < 0.01 for within-group increases from the first to third trimester analyzed by Wilcoxon’s matched-pairs signed rank tests.

We quantified the angiotensin metabolites upstream (Ang I) and downstream (Ang III and Ang IV, generated via N-terminal metabolism; and Ang-1-5 and Ang-1-7, generated via C-terminal metabolism) from Ang II (Figure 2A–D, Table 2). In the NT group, the concentrations of Ang II only modestly increased across gestation (*p* < 0.05), although the formation of Ang I robustly increased across pregnancy (*p* < 0.0001; Figure 2A,B, Table 2). In the HYP group, the concentrations of Ang II did not increase at all (*p* = 0.594), but the concentrations of Ang I (non-significantly) increased over gestation (*p* = 0.052; Figure 2A,B, Table 2). The data in Supplementary Table S1 indicate that this effect was predominately influenced by the patients who developed gestational hypertension, where Ang I was modestly increased, in contrast to those who developed pre-eclampsia. There was a relative decrease in Ang II to Ang I over gestation, reflected by a decrease in ACE-S, which was evident in both the NT and HYP groups (Table 2). There was a relative increase in the concentrations of aldosterone relative to Ang II (AA2-R) over gestation in NT patients (*p* < 0.010) but not HYP patients (*p* = 0.141, Table 2). 

The metabolism of Ang II was assessed by examining the concentrations of N- (the sum of the concentrations of Ang III and Ang IV) and C-terminal metabolites (the sum of the concentrations of Ang-1-5 and Ang-1-7), where the C-terminal metabolites are reflective of the activity of the counter-regulatory arm of the RAAS [25]. Concentrations (in pmol/L) of the N- and C-terminal metabolites of Ang II were 10-fold lower than the concentrations of Ang II and Ang I in both the NT and HYP groups (Figure 2B,C, Table 2). Concentrations of Ang-1-7 were below the LLOQ in most patients in the NT group (detected in only 7 and 16 patients in the first and third trimesters, respectively), and in the HYP group (detected in only 7 and 9 patients in the first and third trimesters, respectively), so the median values and IQR for this analyte were less than the LLOQ and are not listed in Table 2. Half the value of the LLOQ (a concentration of 1.5 pmol/L) in the samples with concentrations of Ang-1-7 below the LLOQ was used to calculate the sum of the C-terminal peptides. The concentrations of C- and N-terminal metabolites were not different between NT versus HYP patients in the first or third trimester (Figure 2C,D), but in the NT group, the concentration of C-terminal metabolites significantly increased over gestation (Figure 2D). 

## 3. Discussion

Previous studies have demonstrated that the RAAS is suppressed in pregnancies with pregnancy hypertension, but deviations in the activity of the RAAS compared with normotensive pregnancy have not been comprehensively described. We used RAS Fingerprint to quantify multiple angiotensin metabolites and biomarkers of the RAAS across gestation in pregnant women with or without pregnancy hypertension. The main results from this study, the first to report LC-MS/MS-based RAAS profiles in a prospective study of pregnancy hypertension, are (1) a marked increase across gestation was evident in PRA-S, especially the concentrations of aldosterone, in NT pregnancy, but (2) not in those who developed HYP, in whom PRA-S was largely unchanged after the first trimester of pregnancy and the third-trimester aldosterone concentrations were significantly lower those than the NT group; a subanalysis of the HYP group revealed that this trend was evident in both those who developed gestational hypertension as well as those who developed pre-eclampsia, and were more pronounced in the latter. (3) The increase in the secretion of aldosterone across gestation was proportionally greater than the change in PRA-S or Ang II in both groups, with a more robust effect in the NT group; and (4) C- and N-terminal metabolites, which were present in low concentrations in both groups, generally increased over gestation in the NT group but not the HYP group. Taken together, these findings indicate that normotensive pregnancy is associated with a sustained increased in the concentrations of Ang I (and to a lesser degree, Ang II) over gestation, accompanied by a marked increase in the concentrations of aldosterone and a moderate increase in alternative RAAS metabolites. The predominant features of pregnancies that develop HYP are stalled or waning activation of the RAAS in the second half of pregnancy (accompanied by unchanging levels of angiotensin peptides) and attenuated secretion of aldosterone. 

The comprehensive RAAS profiles generated in our study agree with previous studies reporting low renin and aldosterone levels in normal and hypertensive pregnancy, and extend them to include the detailed activity of angiotensin peptide metabolites over gestation. The concentrations of PRA and aldosterone progressively increased with pregnancy, and can exceed non-pregnant levels by two- to threefold [26,37]. PRA increased early in gestation [38], whereas aldosterone concentrations peaked in the third trimester [39]. In pregnancies that develop hypertension, some studies have shown that PRA is initially elevated, as in normotensive pregnancy, but fails to increase over gestation or decreases to non-pregnant levels [25]. Several studies have demonstrated that concentrations of aldosterone, although reduced compared with normotensive levels, still rise to some degree over pregnancy, even when PRA is declining [25,26]. These studies are consistent with our observations, where PRA-S and aldosterone increased over gestation only in the NT group, but aldosterone remained elevated in the HYP group. Our study did not contain a non-pregnant group for comparison, but the values of the RAAS biomarkers in non-pregnant cohorts generated via the same LC-MS/MS-based methodology are available. The median and IQR reported for PRA-S and Ang II in a cohort of 77 individuals with hypertension was 165.6 (80.6–328.3) pmol/L and 100.9 (56.3–227.0) pmol/L, respectively [32]. These values are substantially lower than the values of PRA-S and Ang II reported here in the third trimester of the HYP group, suggesting that the activity of the RAAS was still higher compared with the non-pregnant state.

Because pre-eclampsia is associated with a lower volume of plasma [39], the suppression of the RAAS is somewhat paradoxical. In longitudinal studies of pregnancies that developed gestational hypertension or pre-eclampsia, the downward trend of PRA levels over pregnancy correlated with the onset of high blood pressure [25,26,40]. In our study, patients who developed HYP failed to increase the components of the RAS over pregnancy, and generally had lower concentrations of angiotensin peptides in the third trimester. Downregulation of the activity of renin over the course of hypertensive disease during pregnancy suggests a compensatory response to high blood pressure [40], rather than a primary event in the pathogenesis of the disease. An additional explanation for the low activation of the RAAS in hypertensive pregnancies could be the reduced concentration or availability of AGT [41], the substrate for renin. Langer et al. previously made an observation that first-trimester Ang I levels were low compared with those of Ang II (similar to our study), and suggested that Ang II synthesis was not limited by renin but perhaps by AGT [37].

Historical studies by Gant et al. demonstrated that normotensive pregnancies become refractory to the pressor effects of Ang II, presumably to protection against the vasoconstrictive effects of Ang II, where pregnancies that develop hypertension progressively lose this response over the second half of pregnancy [42]. This suggests the factors impacting AT_1_ receptor signaling in pregnancy, such as the presence or absence of AT_2_ receptors [43], metabolic derangements [44], or AT_1_ autoantibody-binding, might facilitate the negative cellular effects of Ang II (the promotion of inflammation [45] or renal complications [46]), which lead to the progression of hypertension. In our cohort, blood pressure was elevated in HYP compared with NT patients in the first trimester, suggesting the presence of underlying vascular dysfunction in this group, potentially caused by previous pre-eclampsia or Type 1 diabetes in this group.

The factors contributing to the secretion of aldosterone in hypertensive pregnancy are complex. Previous studies have reported that concentrations of aldosterone in pregnancy exceeded those expected on the basis of the renin levels [26,42]. In our study, the concentrations of aldosterone relative to Ang II were significantly increased over gestation only in the NT group, but HYP pregnancies still had elevated aldosterone compared with non-pregnant cohorts [32] in the second half of pregnancy, despite no sustained activation of the RAAS. These data suggest that non-Ang II stimulation of aldosterone is a feature of both normotensive and hypertensive pregnancy, but the possibility of the altered sensitivity of the AT_1_ receptors to Ang II in the adrenal cortex in one group compared with another cannot be excluded. Purported non-angiotensin stimulants of aldosterone in pregnancy include ACTH, where ACTH simulates aldosterone not only in normotensive but also in hypertensive pregnancies [47], and VEGF [48]. In pre-eclampsia, the presence of circulating factors, such as the VEGF receptor-binding splice variant, sFlts-1, and AT_1_ autoantibodies, may impair the synthesis or release of aldosterone [49]. In addition, concentrations of aldosterone could be suppressed by excess sodium in pre-eclampsia, resulting from the activation of epithelial sodium channels (ENaC) [50]. Notably, gain-of-function variants in aldosterone synthase (CYP11B2) were preferably present in normotensive versus pre-eclamptic pregnancies, suggesting an evolutionary basis for an active mineralocorticoid receptor system to protect against pre-eclampsia [51]. 

Enhanced concentrations of Ang-1-7 were reported in normal pregnancy and reduced concentrations in pre-eclampsia, where the authors hypothesized that Ang-1-7 may function in healthy pregnancy to oppose the potentially adverse (vasoconstrictive) effects of Ang II [29,52]. Similarly, in a recent study, plasma concentrations of Ang-(1-7) were elevated throughout pregnancy compared with a non-pregnant control group and were reduced in pre-eclampsia [28], suggesting the protective effect of ACE2 against hypertension during pregnancy via the generation of Ang-1-7. In contrast, we report the limited detection of Ang-1-7 in NT and HYP patients (using mass spectrometry, which is the gold standard for the quantification of biological molecules and is a major strength of our study [53,54]); in samples where Ang-1-7 was detected, the concentrations were significantly lower than those reported in other studies using radioimmunoassays [28,29]. Discrepancies in the reported values likely resulted from the different methodologies used for the detection of Ang-1-7, where cross-reactivity in antibody-based assays (as peptide sequences may only differ by one or two amino acids) may explain the artificially high measured concentrations of Ang-1-7 [55]. Notably, the reliability of ELISA for quantifying angiotensin-(1-7) has recently been called into question [56]). Ang-1-5, a C-terminal metabolite of Ang-1-7, is considered to be a component of the alternative RAAS [57,58]. Ang-1-5 was readily detected in our study by mass spectrometry, so we used the sum of the concentrations of Ang-1-7 and Ang-1-6 as an indicator of the alternative RAAS. The concentrations of Ang-1-7 + Ang-1-5 increased over gestation in NT but not HYP pregnancies. However, the concentrations of angiotensin metabolites downstream from Ang II, (either C- or N-terminally degraded) were 10-fold less than the concentrations of Ang II and Ang I. These data suggest that the main bioactive effector of the RAAS is Ang II, and that the modulatory effects of Ang-1-7 in pregnancy are likely to be specific to local tissues [59]. 

Local activity of the RAAS (e.g., kidney, placenta) plays a key role in organs’ adaptation to pregnancy [8]. Studies in non-pregnant animals demonstrated that in tissues with high local activity of the RAAS (e.g., kidney), Ang II is accumulated intracellularly (where binding to the AT1 receptor protects against rapid degradation), and kidney tissue levels exceed plasma levels of Ang II [60]. In a study of mice, kidney, but not plasma, levels of Ang I and Ang-1-7 were elevated in normotensive pregnancy [61], suggesting the importance of a local kidney RAS in the physiology of pregnancy. The placenta is reported to have a local RAS [59], and placenta-derived exosomes and other placental particles may introduce RAS proteins and peptides in into the maternal circulation [62]. In a 2019 review, Lumbers et al. proposed that defective placentation, a key event in the pathophysiology of pre-eclampsia, results in the dysregulated function of the placental RAS, with subsequent effects on the intrarenal RAS that promote hypertension and renal damage, as well as downregulation of the maternal circulating RAS [59]. In our study, we did not identify the tissue source of the systemic angiotensin peptides, but on the basis of previous published studies, we could hypothesize a dysregulated RAS in the placenta and kidney in pregnancies with gestational hypertension or pre-eclampsia. 

Our study has several limitations. We did not differentiate between pre-eclampsia and gestational hypertension due to the small numbers, although a subanalysis revealed that the predominant phenotype of a suppressed RAAS during pregnancy in patients who went on to develop hypertension was similar in both conditions, and was slightly more evident in pre-eclampsia. Pre-eclampsia is a heterogenous disease, where some subtypes may result from a different pathophysiology [6], with a different RAAS profile. Notably, patients with HELLP syndrome did not exhibit the reduction in renin seen in pre-eclamptic patients [63]. Our study did not include postpartum RAAS measurements. Previous studies have demonstrated that blood RAAS values at postpartum return to non-pregnant levels in normotensive and hypertensive pregnancies [37,40,64], but whether the postpartum return to normal is equivalent among the subtypes of pregnancy hypertension has not been fully investigated. Moreover, our study did not control for sodium or volume status, which could affect the concentrations of aldosterone and PRA-S. Although previous studies have indicated that the sensitivity of renin and aldosterone to salt are blunted in pregnancy [42,65], differences in the salt–aldosterone relationships in pregnancy hypertension versus normotensive pregnancies are not well understood. A recent case–control study reported reduced levels of sodium in the serum of pre-eclamptic women [66], but dietary sodium intake has also been linked to adverse outcomes in pre-eclampsia [67]. The effects of salt status on the volume of fluid and the activity of the RAAS may depend on the subtype of pre-eclampsia, e.g., the underlying cause of the disease. In cases where proteinuria is present, low aldosterone or renin levels might be preceded by extracellular volume expansion, low plasma volume, and sodium retention [68], but less is known about the activity of the RAAS and its relationship with fluid balance and salt retention in non-proteinuric pre-eclampsia Future studies might use mathematical models considering various hemodynamic and biochemical features to improve descriptions of disparate pre-eclampsia subtypes [69].

## 4. Methods and Materials

### 4.1. Study Population

All subjects gave informed consent to participate in the study, which was approved by the University of Kentucky’s Institutional Review Board, and all the study’s procedures/methods were performed in accordance with the relevant guidelines and regulations. The cohort comprised pregnant individuals referred to a high-risk maternal fetal medicine clinic. In the current study, patients were without pre-pregnancy hypertension, but otherwise exhibited clinical risk factors for pre-eclampsia [70], where inclusion in the study was on the basis of more than one of the following clinical risk factors: a history of preterm pre-eclampsia, Type 1 or 2 diabetes, or renal or autoimmune disease; or more than two of the following clinical risk factors: nulliparity, obesity (BMI > 30), a family history of pre-eclampsia, Black race, lower income, age 35 years or older, previous pregnancy with a low birthweight or an adverse outcome, or in vitro conception. Patients not meeting these criteria were recruited as controls. The exclusion criteria included age less than 18 or greater than 45 years, multifetal gestation, a history of allergy to aspirin, gastrointestinal bleeding, severe peptic ulcer or liver dysfunction, patients on anticoagulant medications, and women with an anomalous fetus. Body mass index (BMI), as a confounding variable, was addressed by block randomization for body weight (BMI < 30 and ≥30 kg/m^2^) at enrollment.

### 4.2. Study Design, Data Collection, and Groups

The study clinicians in the Maternal Fetal Medicine Unit identified and obtained consent from the patients for the study. Pregnant women between 18 and 45 years of age in the first trimester of pregnancy were recruited by the study clinicians at the time of their routine prenatal visits or ultrasound screening appointments in the first trimester. Patients were followed prospectively for the development of pre-eclampsia, gestational hypertension, and the related pregnancy outcomes. Patients were treated according to the standard clinical guidelines and data collected as part of routine clinical care. Demographic information was collected upon enrollment. Clinical data and maternal blood were collected at routine visits by trained study personnel at the first (11–16 weeks) and third (28–32 weeks) trimesters of pregnancy, and the outcomes were recorded at delivery. Blood pressure was measured following the American Heart Association Guidelines [71], and was determined as the average of two consecutive measurements per arm assessed in a seated, resting position by one observer. Clinical data and outcomes were obtained from electronic medical records (EMR) by a trained clinical coordinator, and input into a REDcap database by the study personnel. Maternal blood was collected during routine prenatal laboratory evaluations. Blood was drawn by trained phlebotomists from patients in a seated position after 15 min of rest, and transferred into red-topped serum tubes. The study personnel collected the whole blood, which was allowed to rest at room temperature for 60 min, followed by centrifugation, transferring them into 200 μL single-use aliquots, and barcoding. Samples were stored by the study personnel at −80 °C until analysis for angiotensin peptides and aldosterone. 

The development of pre-eclampsia or gestational hypertension was the primary endpoint. Pregnancy outcomes were determined after delivery by trained clinical staff. Pre-eclampsia was diagnosed according to the guidelines of the American College of Gynecologists [72]: either a systolic blood pressure (SBP) or diastolic blood pressure (DBP) greater than 140 or 90 mmHg, respectively, measured at least 4 h apart after 20 weeks of gestation, and one of the following: proteinuria of 300 mg in 24 h or a urine to protein creatinine ratio of 0.3, elevated serum creatine greater than 1.1 mg/dL, liver function tests (AST/ALT) double the upper limits of normal, platelet counts of <10,000/μL, flash pulmonary edema, or neurologic features including blurry vision or headaches that were not relieved with medication. Gestational hypertension was diagnosed as either an SBP or DBP greater than 140 or 90 mmHg, respectively, after 20 weeks of gestation in women with previously normal blood pressure [72]. The following adverse outcomes were also recorded: gestational diabetes, small for gestational age (defined as fetal weight below the 10th percentile), postpartum hemorrhage, pre-term premature rupture of the membranes (PPROM), pre-term labor or pre-term delivery (<34 weeks), the presence of polyhydramnios, or fetal death.

### 4.3. Quantification of Angiotensin Peptides, Aldosterone, and the Equilibrium-Based Biomarkers PRA-S, ACE-S, and the Aldosterone to Ang II Ratio (AA2-R) in Serum Using RAS Fingerprint^TM^

The analysis was performed by Attoquant Diagnostics (Vienna, Austria). Serum concentrations of six angiotensin peptides (Ang I, Ang II, Ang III, Ang IV, Ang-1-5, and Ang-1-7) and aldosterone were quantified from serum samples obtained from the study patients by LC-MS/MS following controlled ex vivo equilibration (i.e., equilibrium analysis), which has been described previously [73,74,75,76]. Equilibrium analysis generates a “snapshot” of RAAS activity in a sample that reflects the biochemical features of the circulating RAAS, based on the principle that all the components required to generate angiotensin metabolites in vivo are present in plasma. Briefly, serum samples were incubated at 37 °C for 1 h to generate a controlled equilibrium, followed by stabilization through the addition of an enzyme inhibitor cocktail. The samples were spiked with stable isotope-labeled internal standards at 200 pg/mL for each angiotensin metabolite. The samples were subjected to C-18-based solid-phase extraction, followed by LC-MS/MS analysis using a reversed-phase analytical column (Acquity UPLC C18, Waters, Milford, MA, USA) operating in line with a Xevo TQ-S triple quadruple mass spectrometer in the multiple reaction monitoring mode (Waters, Milford, MA, USA). Internal standards were used to correct for peptide recovery during the samples’ preparation procedure for each analyte in each individual sample. Analyte concentrations were determined using MassLynx/Target/Lynx software Version 4.2 (Waters, Milford, MA, USA) via integration of the total ion chromatogram obtained from the sum of the quantifier transitions that had been optimized for sensitivity and specificity, and the integrated signals that exceeded a signal-to noise ratio of 10. The concentrations of each analyte were determined via linear calibration and are reported in pmol/L. Batch performance was evaluated on the basis of the calculated levels of the calibrator and the quality control samples (these must be within 15% of the nominal concentration); the lowest calibrator (the lower limit of quantification, LLOQ) must be within 20% of the nominal concentration; at least 67% of all quality control samples and calibrators must comply. The lower limits of quantification (LLOQs) for each peptide were as follows: Angiotensin I, 5 pmol/L; Angiotensin II, 4 pmol/L; Angiotensin III, 4 pmol/L; Angiotensin IV, 2 pmol/L; Angiotensin-(1-5), 2 pmol/L; Angiotensin-(1-7), 3 pmol/L; Aldosterone, 13 pmol/L. For values below the LLOQ, the means for each analyte and of the equilibrium-based biomarkers were calculated using half the LLOQ [76].

Equilibrium concentrations of Ang I, Ang II, and aldosterone were used to calculate biomarkers reflecting PRA, ACE activity, and the aldosterone to Ang II ratio (AA2-R), as previously described [32,74]. PRA-S: [eqAng I] + [eqAng I] (pmol/L); ACE-S: [eqAng II]/[eqAng I] (pmol/L/pmol/L); AA2-R: [aldosterone]/[eqAng II] (pmol/L/pmol/L). PRA-S, ACE-S, and AA2-R have been utilized as biomarkers of the RAAS that correlated with clinical parameters and/or predicted the outcomes in numerous studies, including in humans with hypertension and/or primary aldosteronism [32,77,78,79], heart failure [33,76,80], COVID-19 [81,82], and other conditions [57,83]. 

### 4.4. Statistical Analysis

Statistical analyses were performed with GraphPad Prism version 9.5.1 (San Diego, CA, USA) and SPSS Statistics version 24 (IBM, Armonk, NY, USA). Patients were grouped according to the composite outcomes of the study (development of gestational hypertension or pre-eclampsia) or remaining normotensive. All data were assessed for normality, and the appropriate parametric/non-parametric tests were used. The clinical and demographic data were analyzed for between-group differences (NT versus HYP), where continuous variables (age, BMI, blood pressure, hematocrit, and gestational age) were normally distributed and analyzed using paired *t*-tests, and categorical variables (demographics) were analyzed using chi-square or Fisher’s exact tests. For the serum components of the RAAS, which were not normally distributed, differences were examined between the NT and HYP groups in both the first and third trimester. Mann–Whitney tests were applied for between-group analyses of the independent samples at each time point. The components of the RAAS were examined for changes over time (gestation) within each group. Within-group changes from the first to third trimester were determined using Wilcoxon matched-pairs signed rank tests. A *p*-value less than 0.05 was considered to be statistically significant. 

## 5. Conclusions

In summary, we sought to define the prominent features of the activity of RAAS associated with the development of pregnancy hypertension using a novel methodology for a comprehensive assessment of the RAAS. The concentrations of PRA-S, angiotensin peptides, and aldosterone were not different between the NT and HYP groups in the first trimester, significantly increased over gestation in the NT group only, and tended to be lower in the third trimester in HYP compared with NT patients. We conclude that pregnancies that develop HYP are characterized by stalled or waning activation of the RAAS in the second half of pregnancy (accompanied by unchanging levels of angiotensin peptides) and the attenuated secretion of aldosterone. Clinical applications of this knowledge may inform the detection or treatment of pregnancy hypertension or related adverse outcomes.

## Figures and Tables

**Table 1:** Patient demographics and clinical characteristics of women who developed gestational hypertension or pre-eclampsia (HYP) in pregnancy or who remained normotensive (NT).

Parameter	NT n = 47	HYP n = 27 Patients
Patient demographics, n (%)		
Race		
Caucasian	33 (70)	20 (74)
Black or African-American	7 (15)	4 (15)
Hispanic	6 (13)	2 (7)
Asian	1 (2)	0
Mixed race	0	1 (4)
Primiparous	19 (40)	14 (52)
Previous pre-eclampsia	6 (13)	8 (30)
Type 1 DM	7 (15)	9 (33)
Type 2 DM	5 (11)	2 (7)
Renal disease	1 (2)	0
Autoimmune disease	7 (15)	0
Medications		
Aspirin	22 (47)	18 (67)
Labetalol or nifedipine	0	4 (15)
Antidiabetic agents	12	11
Clinical characteristics, mean ± SD		
Age, years	30 ± 6	28 ± 6
BMI (1st trimester), kg/m^2^	29 ± 7	32 ± 8 ^#^
SBP, mmHg		
First trimester	113 ± 11	122 ± 9 ^###^
Third trimester	115 ± 9	126 ± 10 ^####^
DBP, mmHg		
First trimester	73 ± 8	80 ± 6 ^###^
Third trimester	73 ± 6	81 ± 6 ^####^
Hematocrit, %		
Firstt Trimester	37.8 ± 3.4	38.2 ± 2.5
Third trimester	34.9 ± 2.9	34.3 ± 2.7
Gestational age at delivery, weeks	38.6 ± 1.5	36.8 ± 1.6 ^####^
Outcomes, n (%)		
Gestational hypertension	0	23 (85)
Pre-eclampsia	0	6 (22)
Gestational diabetes	3 (6)	4 (15)
PPROM	3 (6)	1 (4)
Polyhydramnios	4 (9)	2 (7)
IUGR	2 (5)	0
Pre-term labor or delivery	3 (6)	0
Postpartum hemorrhage	7 (15)	4 (15)

Abbreviations: NT, normotensive pregnancy; HYP, patients developed gestational hypertension or pre-eclampsia; DM, diabetes mellitus; BMI, body mass index; SBP, systolic blood pressure; DBP, diastolic blood pressure; PPROM, pre-term premature rupture of the membranes; IUGR, intrauterine growth restriction. ^#^, *p* < 0.05; ^###^, *p* < 0.001; ^####^, *p* < 0.0001 as analyzed by *t*-tests.

**Table 2:** Serum concentrations of the components of the RAAS in the first and third trimesters of pregnancy in women without pre-pregnancy hypertension who subsequently developed gestational hypertension or pre-eclampsia (HYP) during pregnancy or remained normotensive (NT).

RAAS Component	NT	HYP	*p* Value ^@^
	n = 47	n = 27	
Ang I, pmol/L			
First trimester	72.7 (45.9–112.1)	61.6 (36.2–104.1)	0.645
Third trimester	115.6 (69.2–172.7) ****	71.6 (51.2–145.1)	0.08
Ang II, pmol/L			
First trimester	165.3 (110.5–197.0)	143.9 (76.3–211.7)	0.404
Third trimester	180.9 (130.8–290.2) *	141.9 (87.8–244.5)	0.053
Ang III, pmol/L			
First trimester	4.3 (2.0–9.2)	2.0 (2.0–7.3)	0.602
Third trimester	5.9 (2.0–10.1)	2.0 (2.0–4.9)	0.012
Ang IV, pmol/L			
First trimester	8.6 (5.3–12.9)	5.8 (3.4–10.1)	0.107
Third trimester	10.1 (5.2–14.0)	7.4 (4.6–11.9)	0.149
Ang-1-5, pmol/L			
First trimester	5.3 (3.4–7.8)	4.3 (3.0–8.3)	0.571
Third trimester	7.7 (5.3–10.9) **	5.2 (3.3–8.2)	0.084
Aldosterone, pmol/L			
First trimester	386.1 (226.2–626.2)	290.2 (181.3–791.0)	0.68
Third trimester	970.5 (468.3–1586.0) ****	492.5 (260.3–826.9)	0.003
PRA-S, pmol/L			
First trimester	243.2 (157.5–309.1)	194.3 (126.8–341.7)	0.426
Third trimester	307.9 (214.7–474.8) ***	223.7 (143.4–343.2)	0.067
ACE-S, pmol/L/pmol/L			
First trimester	2.16 (1.83–2.98)	2.27 (1.79–3.21)	0.703
Third trimester	1.78 (1.30–2.08) ****	1.83 (1.45–2.41) **	0.534
Aldosterone-Ang II ratio (AA2-R), pmol/L/pmol/L			
First trimester	2.07 (1.63–3.33)	2.25 (0.90–4.67)	0.929
Third trimester	4.25 (3.20–7.71) ****	3.38 (2.04–6.27)	0.161

Data are the median and interquartile range (IQR). ^@^, NT versus HYP within trimester, analyzed by the Mann–Whitney test. *, *p* < 0.05, **, *p* < 0.01, ***, *p* < 0.001, ****, *p* < 0.0001 for the third trimester vs. the first trimester within groups, analyzed by Wilcoxon’s signed rank test. Abbreviations: PRA-S, surrogate biomarker for plasma renin activity, calculated from [Ang I] + [Ang II]; ACE-S, surrogate biomarker for ACE activity, calculated from [Ang II]/[Ang I].

## Data Availability

Data may be made available by the investigators upon reasonable request to the corresponding author: robin.shoemaker@uky.edu.

## Supplementary Materials

The following supporting information can be downloaded at: https://www.mdpi.com/article/10.3390/ijms241612728/s1.
