# Supplementary material for: Activation of the Renin–Angiotensin–Aldosterone System Is Attenuated in Hypertensive Compared with Normotensive Pregnancy
Source: Int J Mol Sci. 2023 Aug 12;24(16):12728. doi: 10.3390/ijms241612728 (PMC10454898; doi:10.3390/ijms241612728)
Supplement: Supplementary file 1 [file ijms-24-12728-s001.zip › ijms-2509646-supplementary.pdf]

RAAS Component	Gestational hypertension N=21	Pre-eclampsia N=6	P value@
Ang I, pmol/L			
1st Trimester	75.5 (40.9 – 102.4)	52.4 (3.4 – 506.1)	0.747
3rd Trimester	81.9 (55.6 – 145.1)*	55.4 (4.7 – 273.5)	0.345
Ang II, pmol/L			
1st Trimester	143.9 (94.0 – 192.9)	122.5 (17.8 – 1077.0)	0.887
3rd Trimester	150.1 (88.8 – 244.5)	120.0 (16.5 – 696.4)	0.441
Ang III, pmol/L			
1st Trimester	2.0 (2.0 – 7.3)	3.2 (2.0 – 42.7)	0.842
3rd Trimester	2.0 (2.0 – 4.9)	2.6 (2.0 – 11.5)	0.988
Ang IV, pmol/L			
1st Trimester	5.3 (3.8 – 9.5)	7.9 (1.0 – 76.2)	0.875
3rd Trimester	8.0 (4.9 – 11.9)	5.8 (3.5 – 21.2)	0.811
Ang-1-5, pmol/L			
1st Trimester	4.2 (3.0 – 7.3)	6.9 (1.0 – 36.5)	0.369
3rd Trimester	4.8 (3.7 – 7.9)	6.9 (1.0 – 28.1)	0.449
Aldosterone, pmol/L			
1st Trimester	269.6 (181.3 – 791.0)	351.0 (9.7 – 839.7)	0.843
3rd Trimester	492.5 (264.1 – 826.9)	440.2 (118.4 – 1103.0)	0.798
PRA-S, pmol/L			
1st Trimester	194.3 (130.0 – 277.9)	174.8 (21.2 – 1583.0)	0.887
3rd Trimester	232.0 (145.2 – 343.2)	175.3 (21.2 – 969.9)	0.408
ACE-S, pmol/L / pmol/L			
1st Trimester	2.30 (1.67 – 3.21)	2.24 (1.79 – 5.27)	0.798
3rd Trimester	1.58 (1.45 – 2.19)**	2.19 (0.99 – 3.47)	0.339
Aldosterone-Ang II ratio (AA2-R), pmol/L / pmol/L			
1st Trimester	2.48 (1.13 – 4.67)	1.72 (0.55 – 5.17)	0.345
3rd Trimester	3.38 (2.04 – 6.12)	3.42 (1.10 – 9.65)	0.974

Supplementary Table S1. Serum concentrations of angiotensin (Ang) peptides, aldosterone, and the biomarkers PRA-S, ACE-S, and AA2-R at equilibrium determined by liquid chromatography with tandem mass spectrometry (LC-MS/MS) in the first and third trimesters of pregnancy in women that developed gestational hypertension or pre-eclampsia in pregnancy. Data are median and interquartile range (IQR). @, Gestational hypertension versus pre-eclampsia within trimester, analyzed by Mann-Whitney test. *, P<0.05, and **, P<0.01 for third trimester vs first trimester within group analyzed by Wilcoxon signed rank test. Abbreviations: PRA-S: surrogate biomarker for plasma renin activity, calculated from ([Ang I] + [Ang II]); ACE-S: surrogate biomarker for ACE activity, calculated from ([Ang II] / [Ang I]).
